# EEG correlates of developmental dyslexia: a systematic review

**DOI:** 10.1007/s11881-022-00273-1

**Published:** 2022-11-22

**Authors:** Elisa Cainelli, Luca Vedovelli, Barbara Carretti, Patrizia Bisiacchi

**Affiliations:** 1 Department of General Psychology, University of Padova, Via Venezia, 8, 35133 Padua, Italy; 2 Unit of Biostatistics, Epidemiology, and Public Health, Department of Cardiac, Thoracic, Vascular Sciences, and Public Health, University of Padova, Padua, Italy; 3 Padova Neuroscience Centre, PNC, Padua, Italy

**Keywords:** Neurophysiology, Spectra, Oscillations, Connectivity, Reading, Learning

## Abstract

Dyslexia is one of the most studied learning disorders. Despite this, its biological basis and main causes are still not fully understood. Electroencephalography (EEG) could be a powerful tool in identifying the underlying mechanisms, but knowledge of the EEG correlates of developmental dyslexia (DD) remains elusive. We aimed to systematically review the evidence on EEG correlates of DD and establish their quality. In July 2021, we carried out an online search of the PubMed and Scopus databases to identify published articles on EEG correlates in children with dyslexia aged 6 to 12 years without comorbidities. We follow the PRISMA guidelines and assess the quality using the Appraisal Tool questionnaire. Our final analysis included 49 studies (14% high quality, 63% medium, 20% low, and 2% very low). Studies differed greatly in methodology, making a summary of their results challenging. However, some points came to light. Even at rest, children with dyslexia and children in the control group exhibited differences in several EEG measures, particularly in theta and alpha frequencies; these frequencies appear to be associated with learning performance. During reading-related tasks, the differences between dyslexic and control children seem more localized in the left temporoparietal sites. The EEG activity of children with dyslexia and children in the control group differed in many aspects, both at rest and during reading-related tasks. Our data are compatible with neuroimaging studies in the same diagnostic group and expand the literature by offering new insights into functional significance.

**Supplementary Information:**

The online version contains supplementary material available at 10.1007/s11881-022-00273-1.

## Definition and cognitive mechanisms underlying developmental dyslexia

DD manifests as an unexpected difficulty in acquiring reading skills despite adequate education, intelligence, and sociocultural opportunities and without obvious sensory deficits. Depending on the characteristics of the language, accuracy and/or fluency can be affected (Diamanti et al., 2018). According to the *Diagnostic and Statistical Manual for Mental Disorders* (5th ed.; *DSM-5*; American Psychiatric Association 2013), the incidence of specific learning disorders, including DD, ranges from 5 to 12%. Following *DSM-5*, the main four criteria for diagnosing DD include the presence of difficulties in learning to read that have persisted for at least 6 months despite additional help or targeted instruction being provided. These difficulties interfere with everyday activities (such as academic achievement) and are well below the age-expected level (defined as performance below 1.5 average *SD*). Reading problems manifest upon admission into the school system and are not explained by other impairments such as intellectual disabilities, sensory, or neurological problems. By involving reading acquisition, a central skill in most school systems, DD is associated with many negative school outcomes, including reduced educational attainment and academic self-efficacy (Elgendi et al., 2021). Due to its developmental nature, DD persists until adulthood, with consequences also in the work context (Nalavany et al., 2018).

As largely demonstrated, learning to read involves multiple processes ranging from cognitive and linguistic abilities to visual and attentional processes. Although, in the past, the effort was to identify the single causal mechanism of dyslexia, more recently, it has been recognized that variable patterns of weakness can contribute to reading difficulty in children (O’Brien & Yeatman, 2021). Research on developmental dyslexia has indeed documented deficits in vision (e.g., Stein & Walsh, 1997), attention (e.g., Vidyasagar & Pammer, 2010), auditory and temporal processes (e.g., Vandermosten et al., 2010), and phonology and language (e.g., Hulme et al., 2015). In addition, weaknesses in executive functions, particularly in working memory, have been reported (e.g., Lonergan et al., 2019).

Using neuroimaging techniques such as functional magnetic resonance imaging (fMRI), researchers have identified brain circuits crucially involved in typical and dyslexic reading. A coarse neuroanatomical model of reading and DD has proposed abnormal brain activation occurs in dyslexic readers in the left posterior temporoparietal cortex (middle temporal gyrus, superior temporal gyrus, supramarginal gyrus, and angular gyrus), the left occipitotemporal cortex (inferior temporal gyrus and fusiform gyrus), and the left frontal cortex (inferior frontal gyrus and precentral gyrusHancock et al., 2017; Martin et al., 2016; Richlan, 2020; Richlan et al., 2009).

However, although there are great improvements in comprehending the involved neuroanatomical circuits, little evidence exists to show that fundamental brain processes are affected and how the brain compensates for those disruptions.

Although the spatial resolution is lower compared to fMRI, electrical signals allow for exploring networks with temporal dynamics that functionally do not completely overlap with their fMRI counterparts. Many electrophysiological studies have provided evidence for basic perceptual deficits in DD. Abnormal event-related potentials (ERPs) for auditory and visual processing of speech and non-speech stimuli were found in both children and adults with dyslexia (for example, Bishop, 2007; Hämäläinen et al., 2013; Heim & Keil, 2004; Schulte-Körne & Bruder, 2010). ERPs are measures of electrical activity driven by changes in cognitive processing that are usually time locked to stimuli and could be defined as a measure of the flow of sensory-related and action-related information in neuronal networks of the brain (even if some evidence suggests that some ERP components might be generated by stimulus-induced changes in ongoing brain dynamics (Penny et al., 2002). ERPs are extrapolated from the electroencephalogram (EEG), which, as a whole, provides insight into functional brain organization through the patterns of different brain oscillations. EEG shows overlapping electrical oscillation rhythms representing spontaneous activities in resting states with eyes open and closed. In response to stimuli, EEG rhythms react by synchronizing and desynchronizing, which does not represent signal processing per se*,* but rather a modulation of the information flow in the brain following stimulation.

Although EEG rhythms have been discarded and ignored for years, considered a noisy background activity, the appearance of new methods in recent years has allowed the latter to face its renascence. The spectral power in the different frequency bands is the first and simpler source of information we can obtain from quantitative analysis of EEG, despite the different analysis techniques. It is determined by the synchronous activity of oscillating networks of neurons, and it reflects crucial aspects of processing information in the brain (Buzsáki & Draguhn, 2004). Phase synchronization of brain oscillations across spatially distinct brain regions has been suggested to be an important neuronal communication mechanism by dynamically linking neurons into functional networks (Womelsdorf et al., 2007). Under stimulation, endogenous oscillations phase reset their activity to the rhythmic information in the input, synchronizing cell activity so that peaks in excitation co-occur with stimulus delivery, thereby enhancing neural processing (Canolty et al., 2006; Lakatos et al., 2005). The different frequencies at which the networks oscillate have been divided into five groups—delta (0.5–4 Hz), theta (4–7 Hz), alpha (8–12 Hz), beta (13–30 Hz), and gamma (> 30 Hz)—with different functional meanings and involvement in a variety of perceptual, sensorimotor, and cognitive operations. Alpha-band oscillations are the dominant oscillations in the human brain with an active role in information processing and a possible inhibitory function (Klimesch, 2012). Abundant during sleep, in the awake state delta is associated with functional cortical deafferentation or inhibition of the sensory input that interferes with internal concentration (Harmony, 2013). The existence of several beta rhythms with different frequencies, topographies, and different functional properties presumes no single neuronal mechanism for their generation (Kropotov, 2009). Finally, it has been shown that gamma band activity plays a crucial role in several cognitive tasks; moreover, it seems to interact with the activity in other frequency bands: in speech tasks, gamma interacts with theta, which accounts for syllabic perception, becoming crucial in processing linguistic stimuli (Giraud & Poeppel, 2012). The current hypothesis is that alterations in the oscillatory patterns of EEG play a critical role in the maintenance of brain functions and, consequently, may offer crucial information about brain functions.

This work aims to systematically review the literature on the EEG correlates of DD. We will exclude the broad category of ERPs, given the different functional meanings and also considering the presence in the literature of many reviews about them (for example, Bishop, 2007; Hämäläinen et al., 2013; Heim & Keil, 2004; Schulte-Körne & Bruder, 2010). We focused on children who received the first diagnosis of dyslexia to analyze this problem. Several reports in the literature suggest that the first diagnosis of learning disabilities is more frequent during primary school (e.g., Arrhenius et al., 2021). For this reason, we focused on the age range of 6–12 years. We intended to identify and retrieve international evidence, establish the quality of that evidence, address any uncertainty, and evaluate and synthesize the results. We hope that conflicting evidence could lead to further research.

## Methods

### Protocol and registration

We performed a systematic review of published journal articles on the correlates of EEG in DD, following the PRISMA guidelines (Page et al., 2021). The study protocol has been registered and is publicly available at https://osf.io/4yz7j, where the resources obtained from this study are also available.

### Eligibility criteria

#### Types of studies

Case series and case–control studies investigating the correlates of EEG in DD were included. Participants in each study had a diagnosis of dyslexia according to current diagnostic manuals (e.g., ICD, DSM) and/or national guidelines. No publication date or publication status restrictions were imposed. Only English studies were included.

#### Types of participants

Participants aged 6 to 12 years with DD (i.e., not acquired) were included. To limit the exclusion of works, we included those works with broader age ranges but in which the results differentiated for age. That means that works that include older children but allow for extrapolating specific results on 6–12 age ranges have been included. Comorbidities were considered exclusion criteria; in studies in which patients with comorbidities were also involved, but patients without were also present, only the results for the latter were considered.

#### Types of outcome measures

Except for ERPs, all EEG methodologies were included (in the supplementary materials, a description of EEG measures is reported).

### Information sources

We conducted our search in July 2021 using PubMed and SCOPUS (Elsevier API) bibliographic databases, which include most of the EMBASE database (https://www.elsevier.com/solutions/embase-biomedical-research). The search was conducted using the following string: dyslexia AND (children OR developmental OR pediatric OR paediatric) AND EEG. This string returned 261 results in Scopus and 458 in PubMed. The final search results were exported to store and remove duplicates in the Mendeley bibliographic software package. There was only an internal duplicate within the PubMed database. Internal and external duplicates between the databases were removed from the list. The electronic database search was supplemented by screening the reference lists of each retrieved paper and scanning relevant reviews, obtaining two additional works. In total, 560 results were selected.

### Study selection and data collection process

The eligibility assessment was performed independently and standardized in an unblinded manner by two reviewers (E. C. and L. V.). A third reviewer resolved disagreements between reviewers (P. B. or B. C.). We developed a data extraction sheet that captured relevant information on key study characteristics and all EEG techniques used to investigate DD. Studies have been double coded.

### Data items

The following information was collected from the records: year of publication, groups (e.g., patients with DD, healthy controls, or controls with other clinical characteristics), sample sizes, age at testing, criteria for defining the diagnosis of DD, EEG methodology, experimental conditions, supplementary neuropsychological/cognitive/achievements measures, EEG results, and correlation between EEG findings and supplementary measures.

### Risk of bias in individual studies

The risk of bias at the study level was assessed by two reviewers (E. C. and L. V.) using the Appraisal Tool for Cross‐Sectional Studies (AXIS; Downes et al., 2016). This 20‐item tool was developed in response to the increase in cross‐sectional studies that inform evidence-based medicine and the consequent importance of ensuring that these studies are of high quality and low bias. AXIS assesses the quality of cross‐sectional studies based on the following criteria: clarity of objectives/objectives and target population; appropriate study design and sampling framework; justification for sample size; measures taken to address non-respondents and the potential for response bias; risk factors and outcome variables measured in the study; clarity of methods and statistical approach; appropriate presentation of results, including internal consistency; justified discussion points and conclusion; discussion of limitations; and identification of ethical approval and conflicts of interest. The scoring system conforms to a “yes,” “no,” or “do not know/comment” design. We classified the studies into four quality categories based on the number of “yes” answers for each of the 20 questions included in the AXIS tool as follows (Bull et al., 2019): “high” (more than 15 positive answers), “medium” (between 10 and 15), “low” (between 5 and 9), and “very low” (equal or less than 4). The overall quality categories of the studies are reported in Table 1.

**Table 1:** The cumulative quality score of all studies obtained from the AXIS questionnaire

**Quality**	*N* = 49^1^
High	7 (14%)
Medium	31 (63%)
Low	10 (20%)
Very low	1 (2.0%)

^1^*n* (%)

## Results

### Study selection

**Fig. 1:**
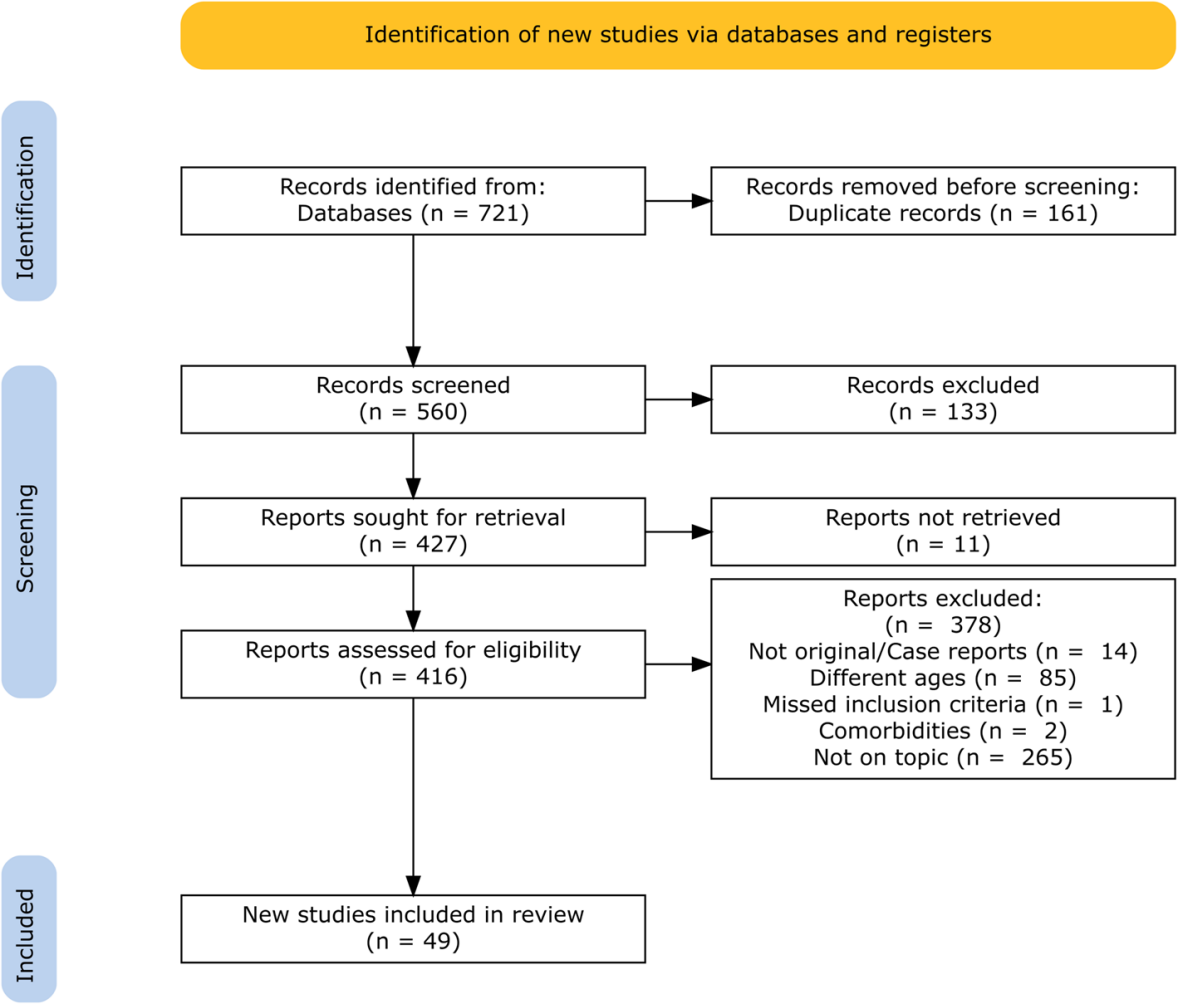
Study workflow

Figure 1 summarizes the following workflow (Haddaway & McGuinness, 2020). The 560 results were screened based on the title of the articles, and 133 were excluded for not being neurophysiological studies investigating DD, for not being original research (reviews, meta-analyses, abstracts, or proceedings), or for not being in English. The full texts of the remaining 427 articles were screened, and further exclusion criteria excluded 378 additional articles. Articles were excluded based on not being original quantitative research or case reports (*n* = 14), involving participants outside the age range selected without differentiation between ages (*n* = 85), diagnosing dyslexia in a way not defined according to inclusion criteria (*n* = 1), involving participants with comorbidities (*n* = 2), not focusing on EEG in DD (*n* = 265), and being irretrievable (*n* = 11). The final analysis included 49 studies.

### Quality of studies

**Fig. 2:**
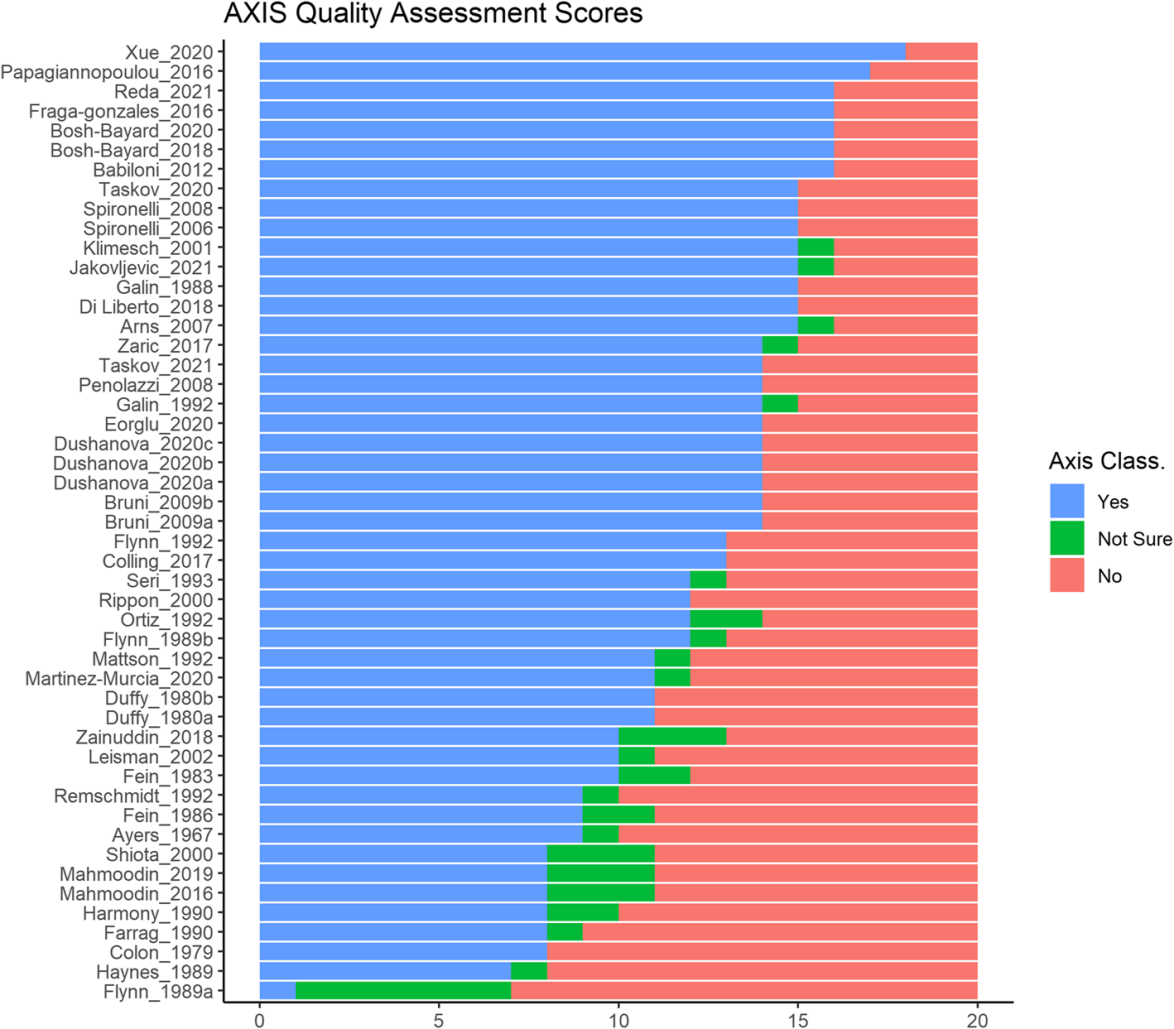
“Yes,” “no,” and “not sure” responses to the 20 items of the AXIS questionnaire for each included study

Individual scores for each included study are reported in Fig. 2.

**Fig. 3:**
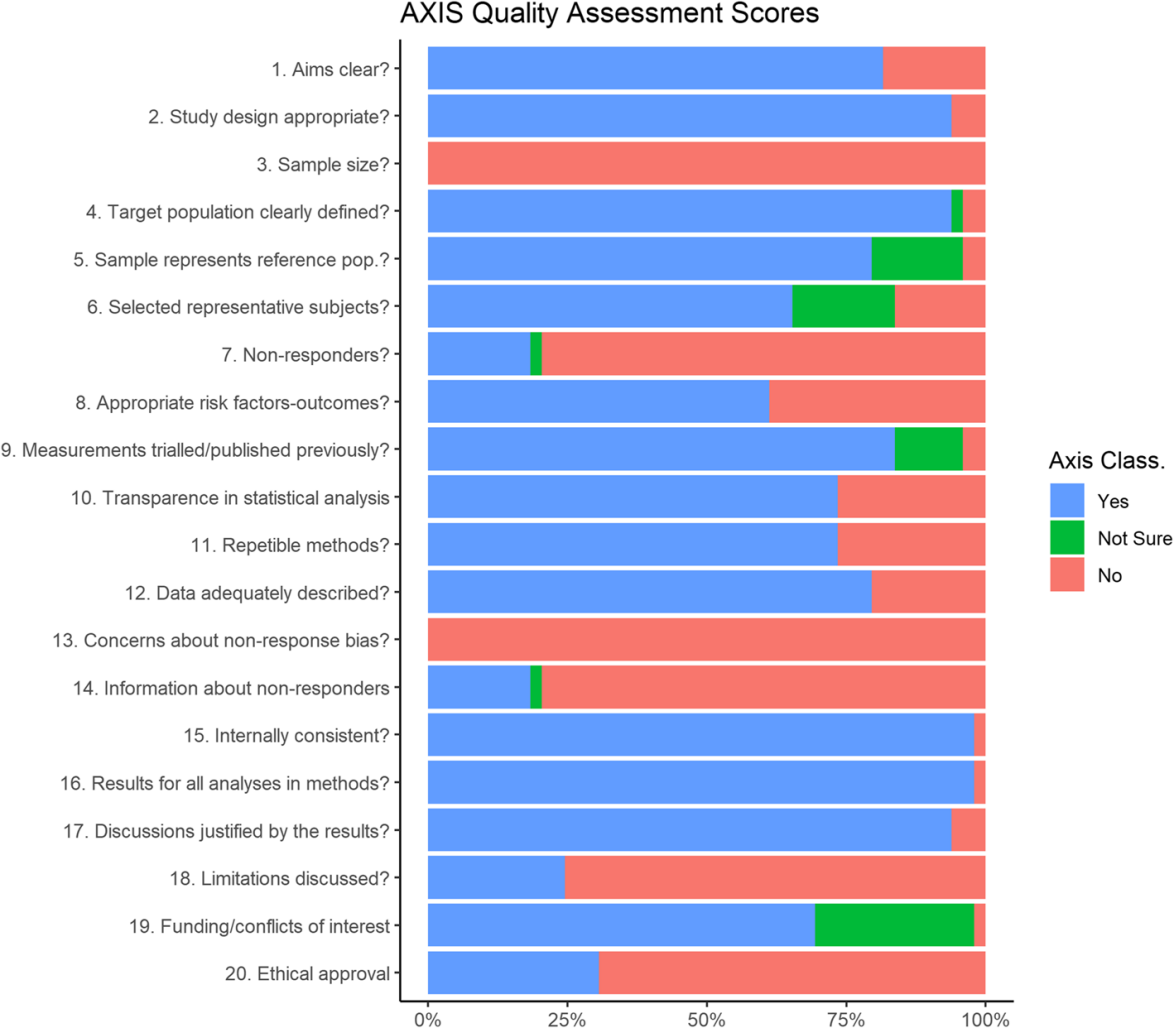
Percentage of “yes,” “no,” and “not sure” responses obtained in the sample of studies for each question of the AXIS questionnaire

The cumulative quality score of all studies relative to the AXIS questionnaire is reported in Table 1. Only 14% were classified as high-quality level (> 15 positive answers), while the majority (63%) fell into the medium level. Finally, the quality of 20% was considered low and, for 1 study, very low. The most common vulnerabilities are the sample size estimate, ethical approval information, and missing data management. The percentage of responses for each question is shown in Fig. 3.

Some elements of the AXIS on the study design are positive in all studies due to part of the inclusion criteria.

### Typology of the studies

Although EEG is a well-known methodology, we lack normative data to interpret quantitative findings, particularly for relatively new methodologies such as connectivity. Therefore, all studies are case–control comparisons of children with dyslexia and control children without the disorder. Only three studies did not compare children with dyslexia with controls. Still, children with dyslexia with nonspecific reading delay (Bosch-Bayard, 2018 2020) and children with dyslexia who have poor reading ability were compared to children with dyslexia who have capable reading ability (Mahmoodin 2016).

The studies could be at rest (resting state) or during cognitive stimulation recorded by EEG: 12 out of the 49 studies used both approaches. Eighteen of the 49 studies were published before 2000. Table 2 reports all the included studies and their main characteristics (authors, year of publication, sample size, age of the children at the evaluation, language used, methodology, and study quality following the AXIS questionnaire).

**Table 2:** Authors, year of publication, sample size, age of the children at the evaluation, language used, methodology (type of EEG analysis, at rest/during a task condition), and study quality following the AXIS questionnaire of all studies included in the revision

Authors, year	Sample size	Age	Language	Methods	Quality
Arns et al. (2007)	19 DD 19 C	DD: 10.33 (8.0–15.98) C: 10.34 (8.0–16.03)	Dutch	Spectral power At rest	Medium
Ayers and Torres (1967)	129 DD 47 C 31 R	DD: 104.1 (95–122) C: 107.2 (96–114) R: 106.9 (100–134)	English	Visual inspection At rest	Low
Babiloni et al. (2012)	26 DD 11 C	DD: 11 years ± 0.5 C: 11 years ± 0.7	Italian	Spectral power Loreta At rest	High
Bosch-Bayard et al. (2018)	169 DD 36 NSRD	DD: 9.1 (7–15) NSRD: 9.8 (7–15)	Italian	Spectral power Vareta At rest	High
Bosch-Bayard et al. (2020)	184 DD 43 NSRD	DD: 9.1 + 1.9 NSRD: 9.7 + 2.2	Italian	Coherence At rest	High
Bruni et al. (2009a)	16 DD 11 C	DD: 10.8 (8–16) C: 10.1 (7–16)	Italian	Sleep architecture parameters Nocturnal sleep	Medium
Bruni et al. (2009b)	16 DD 11 C	DD: 10.8 (8–16) C: 10.1 (7–16)	Italian	Spectral power Nocturnal sleep	Medium
Colling et al. (2017)	13 DD 10 C	DD: 119.3 (11.3) C: 120.9 (7.9)	English	Spectral power During task	Medium
Colon et al. (1979)	44 DD 49 C	7 and 11.0	Dutch	Spectral power At rest	Low
Di Liberto et al. (2018)	25 DD 45 C	8.6 + 1.5	Australian English	Spectral power During task	Medium
Duffy et al. (1980a)	11 DD 13 C	/	English	Topographic maps At rest During task	Medium
Duffy et al. (1980b)	8 DD 10 C	9.0 and 10.7	English	Topographic maps At rest During task	Medium
Dushanova and Tsokov (2020)	22 DD 21 C	8–9	Bulgarian	Connectivity During task	Medium
Dushanova et al. (2020)	22 DD 21 C	8–9	Bulgarian	Coherence During task	Medium
Dushanova and Tsokov (2021)	22 DD 21 C	8–9	Bulgarian	Connectivity During task	Medium
Eroglu et al. (2022)	16 DD 20 C	8.56 + 1.36	English	Connectivity At rest	Medium
Farrag and El-Behary (1990)	21 DD 16 C 23 R	2nd and 3rd grades of elementary school	Arabic	Visual inspection At rest	Low
Fein et al. (1983)	31 DD 32 C	10–12	English	Spectral power At rest	Medium
Fein et al. (1986)	34 DD 35 C 22 DD 22 C	10–12 9–13	English	Spectral power At rest	Low
Flynn and Deering (1989a)	21 DD 6 C	7.4–10.8	English	Spectral power During task	Very low
Flynn and Deering (1989b)	12 DD disfonetic 4 DD disdeitic 5 DD mixed 6 C	DD1:104 (93–130) DD2:96 (89–110) DD3:101 (90–120) C: 113 (94–128)	English	Spectral power Topographic mapping At rest During task	Medium
Flynn et al. (1992)	27 DD disfonetic 6 DD disedeitic 6 C	8.0–9.11	English	Spectral power At rest During task	Medium
Fraga Gonzales et al. (2016)	26 DD 15 C	DD 8.4 + 0.40 C 8.75 ± 0.31	Dutch	Spectral power Connectivity At rest	High
Galin et al. (1988)	34 DD 35 C 22 DD 22 C	1.10–12 2. 9–13	English	Spectral power At rest During task	Medium
Galin et al. (1992)	34 DD 35 C 22 DD 22 C	1.10–12 2. 9–13	English	Spectral power At rest During task	Medium
Harmony et al. (1990)	Good: 33 Regular: 23 Poor: 17 Very poor: 8	6–12	English	Spectral power At rest	Low
Haynes et al. (1989)	12 DD 12 C	8–12	English	Spectral power During task	Low
Jakovljevi´c et al. (2021)	18 DD 18 C	8–12	Serbian	Spectral power During task	Medium
Klimesch et al. (2001)	8 DD 8 C	DD: 11.6 + 0.5 C: 11.36 + 0.33	German	Spectral power During task	Medium
Leisman (2002)	20 DD 20 C	DD: 7.6 (7–10.9) C: 8.2 (7–11.11)	English	Spectral power Coherence At rest During task	Medium
Mahmoodin et al. (2016)	9 DD 4 poor DD 5 capable DD	7–11	Malay	Spectral power At rest During task	Low
Mahmoodin et al. (2019)	11 poor DD 11 capable DD 11 C	P DD: 8 (7–12) C DD: 8 (7–12) C: 10.5 (7–12)	Malay	Spectral power At rest During task	Low
Martinez-Murcia et al. (2020)	16 DD 32 C	DD: 95.6 + 2.9 C: 94.1 + 3.3	Spanish	Spectral power Connectivity During task	Medium
Mattson et al. (1992)	8 DD 8 arithmetic dis 10 C	DD: 11.3 (9–15) Arit.: 12 (9–15) C: 12.4 (9–15)	English	Spectral power During task	Medium
Ortiz et al. (1992)	14 DD 15 C	DD:10.31 (9–11.7) C: 10.38 (9–12)	Spanish	Spectral power At rest During task	Medium
Papagiannopoulou and Lagopoulos (2016)	21 DD 19 C	DD: 8 + 1.40 C: 8 + 1.64	English	Spectral power At rest	High
Penolazzi et al. (2008)	14 DD 28 C	DD: 10.12 + 2.23 C: 10.01 + 0.18	Italian	Spectral power During task	Medium
Reda et al. (2021)	11 DD 18 C	DD: 11.04 (9–14) C: 11.72 (9–14)	Italian	Sleep architecture parameters Spectral power Nocturnal sleep	High
Remschmidt and Warnke (1992)	30 DD 30 C	DD:10.53 (9–12.11) C: 10.49 (9–12.11)	German	Spectral power At rest During task	Low
Rippon and Brunswick (2000)	19 DD 22 C	DD: 10.66 + 1.46 C: 9.96 + 1.69		Spectral power At rest During task	Medium
Seri and Cerquiglini (1993)	10 DD 10 C	11–12.11	Italian	Spectral power Topographic mapping During task	Medium
Shiota et al. (2000)	7 DD 7 C	7–14	Japanese	Coherence At rest	Low
Spironelli et al. (2006)	10 DD 13 C	DD: 9.25 ± 1.34 C: 9.70 ± 1.17	Italian	Spectral power During task	Medium
Spironelli et al., (2008)	14 DD 28 C	DD: 10.12 + 2.23 C: 10.01 + 0.18	Italian	Spectral power During task	Medium
Taskov and Dushanova (2020)	22 DD 21 C	8–9	Bulgarian	Connectivity During task	Medium
Taskov and Dushanova (2021)	25 DD 21 C	8–9	Bulgarian	Spectral power Connectivity During task	Medium
Xue et al. (2020)	27 DD 40 C	DD: 9.22 + 0.58 C: 9.38 + 0.49	Chinese	Spectral power Connectivity At rest	High
Zainuddin et al. (2018)	17 DD 8 capable DD 8 C	7–12	Malay	Spectral power During task	Medium
Zaric et al. (2017)	18 moderate DD 16 severe DD 20 C	mDD: 9.02 ± 0.45 sDD: 8.92 ± 0.41 C: 8.80 ± 0.38	Dutch	Connectivity During task	Medium

Legend: *C*, controls; *DD*, developmental dyslexia; *NSRD*, non-specific reading delay; *R*, remedial

### Resting-state EEG

We found 24 studies investigating resting-state EEG (RS-EEG): 11 out of 24 studies focused solely on RS-EEG, while the other 13 out of 24 studies performed both an RS-EEG and an EEG during a task (in this section, only the results of the RS-EEG will be reported, whereas the results during a task will be described in the next section).

#### Spectral analysis of the RS-EEG

The methodology used most frequently is spectral analysis, which shows the spectral content in the different frequency bands (delta, 1.5–4 Hz; theta, 4–7 Hz; alpha, 8–12 Hz; beta, 13–40 Hz; gamma, > 40 Hz). The methodology has been used alone (Arns et al., 2007; Bruni et al., 2009; Colon et al., 1979; Fein et al., 1983, 1986; Galin et al., 1988, 1992; Harmony et al., 1990; Mahmoodin et al., 2016, 2019; Papagiannopoulou & Lagopoulos, 2016; Remschmidt & Warnke, 1992; Rippon and Brunswick 2000) or combined with other methods (Babiloni et al., 2012; Bosch-Bayard et al., 2018; Flynn & Deering, 1989a; Flynn et al., 1992; Fraga González et al., 2016; Leisman, 2002; Reda et al., 2021; Xue et al., 2020).

**Fig. 4:**
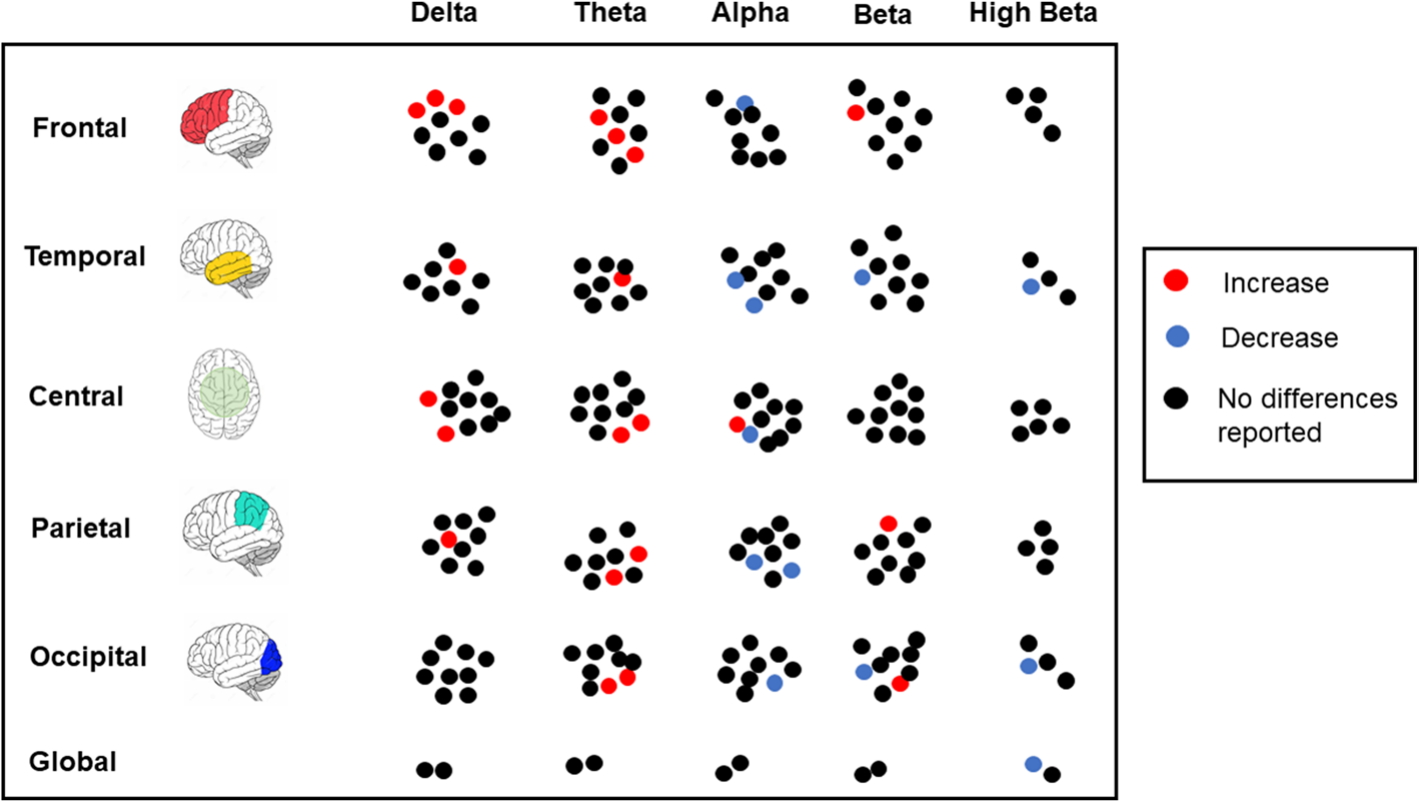
The figure shows the results obtained in the studies using the spectral analysis of RS-EEG. In red the increases and blue the decreases obtained in children with dyslexia compared to controls; in black, if no differences were reported between the groups

Figure 4 shows the results obtained by spectral analysis of RS-EEG. We also reported the non-significant results to render the data more readable. In general, it seems that DD is characterized by an increase in the delta frequency and theta and a reduction in alpha and beta.

#### Other methodologies

Results obtained from other methodologies are less homogeneous, and a summary is not possible; Table 3 reports the results of these studies (Arns et al., 2007; Ayers & Torres, 1967; Babiloni et al., 2012; Bosch-Bayard et al., 2020; Bruni et al., 2009; Duffy et al. 1980a; Eroğlu et al., 2022; Farrag & El-Behary, 1990; Fraga González et al., 2016; Gerald Leisman, 2002; Reda et al., 2021; Shiota et al., 2000; Xue et al., 2020).

**Table 3:** The table below reports authors and year of publication, differences found in DD children from the comparison with controls, and the localization of the difference reported

Author	Measure differences	Localization
Arns et al. (2007)	1. Increased delta coherence 2. Increased alpha and beta coherence	1. Bilateral fronto-central 2. Right fronto-central
Ayers and Torres (1967)	Higher than expected incidence of abnormal electroencephalograms	/
Babiloni et al. (2012)	Lower amplitude in low- and high-frequency alpha rhythms	Parietal, occipital, and temporal cortical sources
Bosch-Bayard et al. (2020)	1. More active hub: the calcarine sulcus is sending information to the right postcentral gyrus, the left paracentral gyrus, the right angular gyrus, and the right supplementary motor area in almost all frequency bands, including delta and theta band 2. Less active hub	1. Left calcarine sulcus 2. Left rolandic operculum
Bruni et al. (2009a)	Increased spindle density during N2 sleep stage	/
Bruni et al. (2009b)	1. Lower number of sleep stage shifts per hour of sleep, percentage of N3, and number of *R* periods 2. Overactivation of the ancillary frontal areas	2. Frontal areas
Duffy et al. (1980a)	Increased alpha activity	Bifrontal areas, left temporal and left posterior regions
Farrag and El-Behary (1990)	Immature EEG tracing by visual inspection	Occipital area
Fraga Gonzales et al. (2016)	Reduced network integration and communication between network nodes in the theta band	/
Leisman (2002)	Greater coherence within the hemisphere	Left parieto-occipital
Reda et al. (2021)	Reduced slow spindles	Occipito-parietal and left fronto-central areas
Shiota et al. (2000)	1. Higher interhemispheric coherence values for alpha and beta 2. Higher interhemispheric coherence values for beta 3. Higher intrahemispheric coherence in alpha	1. Temporal 2. Frontal 3. Central, occipital and parietal
Xue et al. (2020)	Global network deficiencies in the beta band and the network topology was more path-like	/

#### Correlation between resting EEG and reading performance

Some rest studies correlate EEG activity with specific tests performed before or after recordings (Table 4).

**Table 4:** The table reports the results obtained in DD children compared to controls in studies that correlate the EEG activity with specific tests performed before or after the recordings

Author, year	Task	Results
Arns et al. (2007)	Rapid naming of letters (rnl), articulation (ART), phoneme deletion (PD), and spelling (SP)	+ delta coherence with all tests + theta coherence with ART and RNL + alpha with PD and RNL + beta coherence with RNL, PD, and SP
Babiloni et al. (2012)	Two lists of words and pseudowords and reading accuracy	- alpha with a reading time of pseudo-words
Bruni et al. (2009a)	Memory and learning transfer reading test, word and non-word reading test, word, non-word and sentences writing test, WISC-3	+ sigma band in N2 with the word reading and MT reading tests + spindle density with the word reading test
Bruni et al. (2009b)	Memory and learning transfer reading test, word and non-word reading test, word, non-word and sentences writing test; WISC-3; Child Behaviour Checklist (CBCL)	+ A1 index in sleep stage N3 with Verbal IQ, full-scale IQ, and memory and learning transfer reading test + cyclic alternating pattern rate in N3 with verbal IQ
Fraga Gonzales et al. (2016)	3DM reading	No correlation between connectivity (minimum spanning tree) and reading performance
Harmony et al. (1990)	Reading (reading comprehension and oral reading) and writing (copying, dictation, and functional writing)	+ theta in almost all leads in children with minor difficulties, no antecedents, and good socioeconomic status + delta in left frontal and temporal areas (F3, F7, and T3) in children with a poor or very poor evaluation

### EEG during a task

**Fig. 5:**
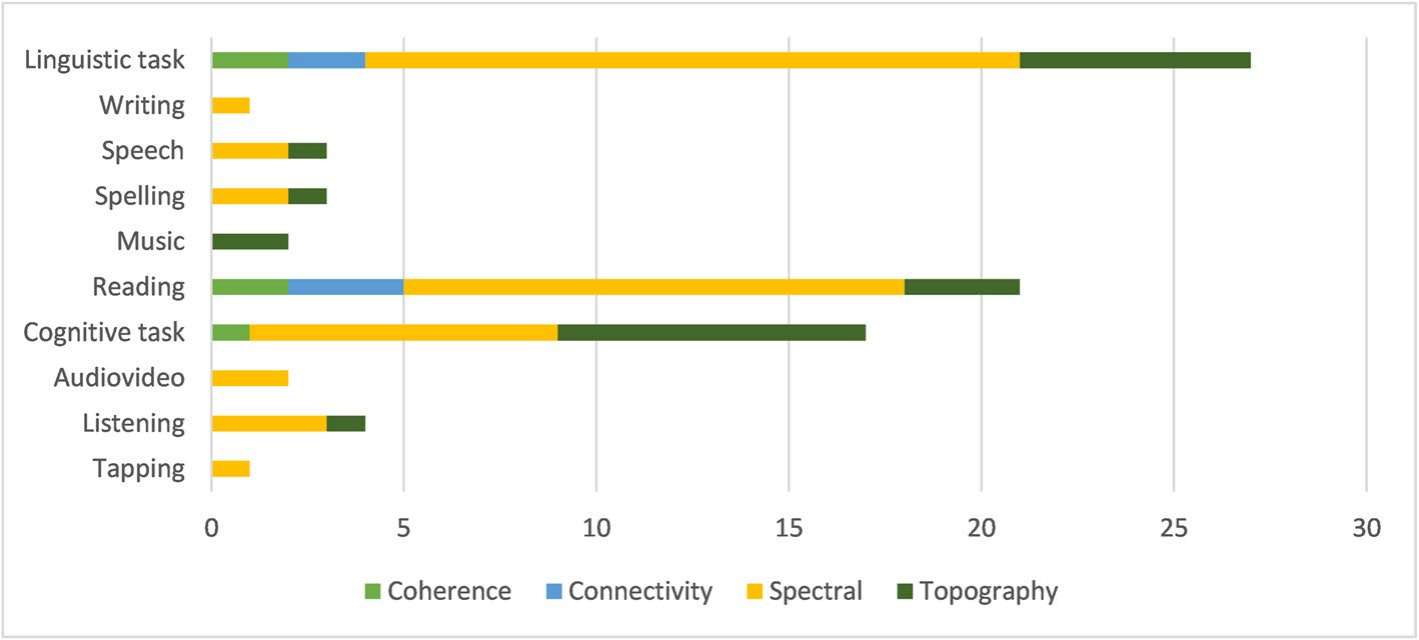
The stimulation task and the methodology applied to the EEG. The figure represents all conditions, so works using multiple tasks and analysis types could be overrepresented

Thirty-two studies explored EEG brain activity during a task. Figure 5 reports the type of stimulation task and the methodology applied to the EEG. Most studies compared the EEG and the performance of DD and control children in linguistic, reading, or cognitive (Go-noGo, attention, reasoning, etc.) tasks (Dushanova & Tsokov, 2020, 2021; Dushanova et al., 2020; Flynn & Deering, 1989a, 1989b; Flynn et al., 1992; Galin et al., 1988, 1992; Jakovljević et al., 2021; Klimesch et al., 2001; Leisman, 2002; Mahmoodin et al., 2016; Ortiz et al., 1992; Penolazzi et al., 2008; Remschmidt & Warnke, 1992; Rippon & Brunswick, 2000; Seri & Cerquiglini, 1993; Spironelli et al., 2006, 2008; Taskov & Dushanova, 2020, 2021; Žarić et al., 2017). A smaller number of other studies evaluate several other tasks: writing, speech, spelling, music, during the vision of an audio story, listening, and tapping (Colling et al., 2017; Di Liberto et al., 2018; Duffy et al., 1980a, 1980b; Flynn et al., 1992; Galin et al., 1992; Haynes et al., 1989; Mahmoodin et al., 2019; Martinez-Murcia et al., 2020; Mattson et al., 1992).

The results are not comparable, given the different tasks and EEG analysis methodologies, but the differences between DD and control children appear mainly localized in the left temporoparietal sites.

Table 5 reports the task used, the differences found in children with dyslexia compared to controls, and the localization findings.

**Table 5:** Authors and year, a brief description of the task used, the differences in EEG brain activity found in dyslexic children compared to controls, and the findings’ localization

Author	Stimulation	Measure differences	Localization
Colling et al. (2017)	- Tapping to every second beat of a metronome pulse - To listening passively to the beat	Preferred phase at 2.4 Hz	Frontal
Di Liberto et al. (2018)	Audio-story while watching the corresponding cartoon	Delta and theta reduction in DD	Right hemisphere
Duffy et al. (1980a)	-Speech: listen and answer questions -Music: listen -Kimura figures instruction	Theta and alpha increase in DD	Left temporal, left posterior quadrant regions, and in the bifrontal area
Dushanova and Tsokov (2020)	To discriminate visually presented words and pseudowords putting a button. Before e post-treatment	Theta, alpha, beta1, and gamma1 strength and betweenness reduction	Left anterior temporal and parietal regions
Dushanova et al. (2020b)	To discriminate auditory presented words and pseudowords putting a button	1. Stronger delta-entrainment for C and visual DD 2. Delta-entrainment deficit for DD 3. Higher delta-entrainment for phonological DD	1. In the left auditory cortex, anterior temporal lobe, frontal, and motor cortices 2. the left anterior temporal lobe, frontal, and the right temporal 3. posterior temporal
Dushanova and Tsokov (2021)	To discriminate words and pseudowords putting a button—before and after training	Theta, alpha, beta degree and betweenness centrality reduction	Left anterior temporal and parietal regions
Flynn and Deering (1989b)	- Reading - Spelling recognition - Drawing a clock	1. Increase in theta for Dyseidetic DD 2. Increase in theta power dyseidetic DD during reading	1. Left temporal-parietal 2. Left mid to posterior temporal and left temporal-parietal
Flynn et al. (1992)	- Listening to a story - Silent reading of text - Oral reading of text - Spelling recognition - Auditory analysis of orally presented words - Drawing a clock	Beta reduction during the reading task	Right occipital-parietal and left temporal-parietal
Galin et al. (1988)	- Kohs block design - Narrative speech	Stronger alpha asymmetry	Temporal leads
Galin et al. (1992)	- Oral and silent reading of easy and hard texts - Listening to a story - Narrative speech	Smaller change in theta and low beta power between tasks	No differences between leads
Haynes et al. (1989)	1. Vigilance condition; 2. listening to a story without an ending that had to be retold; 3. rehearse the story mentally and construct an appropriate ending	Decreased alpha amplitudes in both groups of subjects	No differences between leads
Jakovljevi´c et al. (2021)	Read a story, the text on each slide in different colors	1. Higher values of beta and the broadband EEG (0.5–40 Hz) power while reading in purple 2. Increasing theta range power while reading with the purple overlay	Not reported
Klimesch et al. (2001)	- Reading numbers - Reading words - Reading pseudowords	Large group differences in tonic and phasic lower theta for pseudoword processing	Occipital sites
Leisman (2002)	- Rest-eyes closed - Continuous performance tests - Confrontation naming from the Stanford–Binet - Spache tests (1966) Diagnostic reading tests	1. Greater theta and beta, decreased alpha 2. Lower coherence between hemispheres but greater coherence within the same hemisphere during all tasks	Left parieto-occipital
Mahmoodin et al. (2016)	- Rest eyes closed - Reading a non-word and writing it down based on an auditory cue	Higher beta in capable DD	Frontal (FC6) and parietal (P4) right hemisphere
Mahmoodin et al. (2019)	Listening to amplitude-modulated noise with slow-rhythmic prosodic, syllabic, or the phoneme rates	Higher theta-beta ratio	All leads
Martinez-Murcia et al. (2020)	Listening to amplitude-modulated noise with slow-rhythmic prosodic, syllabic, or the phoneme rates	1. Reduced bilateral connection between electrodes 2. Increased connectivity	1. Temporal lobe 2. F7 electrode
Mattson et al. (1992)	Listening sentences preceded by a warning	1. Reducted 40-Hz activity during verbal task 2. Reducted activity in arithmetic disabled compared to DD and C during the nonverbal task	1. Left hemisphere 2. Right hemisphere
Ortiz et al. (1992)	- Resting-state with eyes closed - Resting condition with eyes open - Auditory phonemic discrimination task	1. Alpha responsiveness during the task 2. High beta decrease during the task	1. Left hemisphere 2. Left posterior quadrant
Penolazzi et al. (2008)	To compare the word pairs based on 1. orthographic 2. phonological 3. semantic criteria	1. Greater overall delta amplitude 2. In the phonological task, larger delta anterior and smaller posterior delta amplitude	1. Anterior sites 2. Left anterior and left posterior
Remschmidt and Warnke (1992)	To mark discriminate letters into letter strings	(3) Faster attenuation of relative alpha power increasing cognitive activation and reading (4) DD did not reveal characteristic focal EEG features	No differences between leads
Rippon and Brunswick (2000)	- Phonological processing task - Visual search task (WISC picture completion)	1. Lack of task-related reduction from resting levels in the amplitude of alpha 2. Marked asymmetry in beta activity 3. In the phonological task, a theta increase	1. Parieto-occipital 2. Parieto-occipital R > L 3. Frontal
Seri and Cerquiglini (1993)	Word length recognition test requiring a gentle right finger-lift response	Lack of desynchronization	1. Right frontal and temporal, left parietal
Spironelli et al. (2006)	- Phonological task (to decide whether the word pairs rhymed) - Semantic task (to decide whether the target word was semantically related to the first)	Delayed theta peak activity and was shifted to the right hemisphere	Right instead of left
Spironelli et al. (2008)	- Phonological task (to decide whether the word pairs rhymed) - Semantic task (to decide whether the target word was semantically related to the first) - Orthographic task (to decide whether word pairs were written in the same case). Pressing a button	1. Significantly greater beta and theta over the right hemisphere during phonological task 2. Greater beta and theta over the left hemisphere during phonological and orthographic tasks 3. Delay in behavioral responses, paralleled by sustained theta	1. Right frontal 2. Left posterior
Taskov and Dushanova (2020)	Reading aloud words—before and after treatment	1. Higher leaf fraction, tree hierarchy, kappa, and smaller diameter (theta–gamma frequency) (less segregated neural network) 2. Reduced degree and betweenness centrality of hubs	1. Globally 2. Superior, middle, and inferior frontal areas in both brain hemispheres
Taskov and Dushanova (2021)	Reading aloud words—before and after treatment	Absent functional connectivity nodes derived from theta frequency network for both conditions	Dorsal medial temporal area, left middle occipitotemporal, parietal
Zaric et al. (2017)	Discriminating word from false font pressing a button	1. Weaker connectivity for words 2. Stronger connectivity for words and false fonts in severe DD	1. Occipital to inferior-temporal 2. From left central to right inferior-temporal and occipital sites

#### Comparing at-rest and during-task conditions

Twelve works were performed both at rest and during the task conditions. Some authors found differences in both conditions, but more pronounced in the task condition (Duffy et al. 1992) or the rest condition (Leisman, 2002), whereas others found differences only during the task condition (Flynn et al., 1992; Galin et al., 1988; Ortiz et al., 1992). Finally, some studies did not clearly report what happened during the at-rest condition (Duffy et al. 1980b; Flynn & Deering, 1989b; Galin et al., 1992; Mahmoodin et al., 2016, 2019; Zainuddin et al., 2018).

### Other kinds of studies

Some studies of EEG in children with dyslexia are not possible to include in the previous paragraphs because of the different natures of the works. We will briefly describe them as follows.

Bosch-Bayard et al. (2018) wanted to find a classification equation that discriminates the two groups with high accuracy. They obtained a discrimination equation that did not participate in the Boder classification algorithm, with a specificity and sensitivity of 0.94 to discriminate DD from the nonspecific reading delay.

Using a statistically based technique, Duffy et al., (1980a, 1980b) searched for rules for the classification of children with dyslexia. They developed classification rules that successfully diagnosed 80 to 90% of the subjects.

Eroglu et al. (2022) investigated possible disturbances in the complexity of EEG signals (connectivity measures) on multiple time scales in people with dyslexia and the potential positive effects of special neurofeedback and multisensory learning treatment. After treatment, the lower complexity of the experimental group increased to the typically developing group on lower and medium temporal scales in all channels. Fein et al. (1983) assessed the test–retest reliability of both absolute and relative spectra. They found excellent absolute and relative power reliability under properly controlled conditions.

Finally, Zainuddin et al. (2018) used a support vector machine algorithm to classify EEG signals from typical, poor, and capable children with dyslexia while writing words and nonwords. Beta and theta-to-beta ratios formed the input features for the classifier. It was found that the best performance of the support vector machine was obtained with 91% overall accuracy when both kernel scale and box constraint were set to 1.

### Differences between dyslexia subtypes

Only a few studies evaluated the differences between dyslexia subtypes. In their works, Bosch-Bayard et al. (2018) and Bosch-Bayard et al. (2020) focused on dyslexia with phonological deficits (dysphonetic) compared with children with nonspecific reading delays. By analyzing the power spectra, in 2018, they found that the DD group had significantly higher activity in the delta and theta bands than the nonspecific reading delays group in the frontal, central, and parietal areas bilaterally. Two years later, using measures of EEG connectivity, they found that the left calcarine sulcus was more active in the DD group, while the left rolandic operculum was more active in the nonspecific reading delays group. Instead, Flynn and Deering (1989a) and Flynn et al. (1992) compared two types of dyslexia: dysphonetic and dyseidetic (with orthographic deficits). They found left temporal differences in children with dyseidetic dyslexia and right parietal-occipital differences for those with dysphonetic dyslexia, supporting predictions derived from a compensation-from-strength model of dyslexia.

## Discussion

We performed a systematic review of the evidence using EEG in DD. Finally, we selected 49 works, both EEG studies at rest and during a task. The articles differed greatly in methodology, which makes a summary of the results challenging. However, some points have come to light. Even at rest, children with dyslexia and children in the control group exhibited differences in several EEG measures, particularly an increase in delta and theta and a reduction in alpha frequencies, without a clear localization. The same frequencies recorded at rest appear to be associated with learning performances. During reading-related tasks, differences between children with dyslexia and children in the control group appear more localized at the left temporoparietal sites, and the spectral frequencies appear differently involved. Theta range remained the frequency band that hosts the main number of differences between children with dyslexia and children in the control group, but some work also found the involvement of the beta and gamma bands.

Current research on electrophysiological correlates of language acquisition could help interpret our data. For example, many studies have been done on speech processing, a cognitive ability strictly associated with reading. Delta, theta, and gamma oscillations have been shown to be specifically engaged by the quasirhythmic properties of speech (Giraud & Poeppel, 2012). Different frequencies account for different properties of the language: the transformation of the auditory signal input into lexical and phrasal units occurs at a very low modulation rate, roughly 1–2 Hz. Frequencies in a slightly higher range (1.5–4; i.e., delta) account for prosodic perception (Ghitza & Greenberg, 2009) and (4–7; i.e., theta) for syllabic perception (Luo & Poeppel, 2007; Poeppel et al., 2008). Higher frequencies (30–40 Hz, the high beta/low gamma band) process stimulus information concurrently with the theta band, lying in a nesting relation such that the phase of theta shapes the properties of gamma (Giraud & Poeppel, 2012). Frequency bands could have similar functions in the reading process. In fact, studies in children, adolescents, and adults with dyslexia converge to identify an atypical auditory neural synchronization of oscillations, suggesting deviant neural processing of both syllabic and phonemic rate information (De Vos et al., 2017; Di Liberto et al., 2018; Lehongre et al. 2011; Lizarazu et al., 2015; Molinaro et al., 2016). It has been proposed that if people with dyslexia parse speech at a frequency slightly higher or lower than the usual frequency rate, their phonemic representations could be abnormal (Ziegler et al., 2009). This anomaly would selectively complicate the grapheme-to-phoneme matching, leaving speech perception and production unaffected. These studies are compatible with the results from ERPs, which revealed altered processing of certain acoustic information relevant to speech perception in individuals with dyslexia, such as frequency changes and temporal patterns (Schulte-Körne & Bruder, 2010).

Competing neurobiological hypotheses alternatively assign a crucial role to higher versus lower frequency bands. It has been suggested that dyslexic people may be less responsive to modulations at specific frequencies that are optimal for phonemic analysis (30 Hz) (Lehongre et al., 2011) or that they may fail to reset gamma activity (Schroeder et al., 2010). Other authors, more in line with the results of our review, emphasized the role of lower frequencies and, in particular, theta oscillations. A deficit in theta is thought to alter low temporal modulation tracking syllable coding and even multisensory processing, with consequences for attention and auditory-visual integration (Goswami, 2011; Ziegler et al., 2009). De Vos and colleagues (De Vos et al., 2017) found in adolescents with DD atypical alpha (reduced) and beta (increased) synchronization. They advocated that the alpha reduction could be related to phonological processing problems. At the same time, the over-synchronization of beta range oscillations could be a compensatory mechanism to improve the processing of phonemic rate information. Although different methodologies and age ranges, we also found numerous abnormalities in alpha and beta frequencies (Duffy et al., 1980a; Dushanova & Tsokov, 2021; Flynn et al., 1992; Galin et al., 1988, 1992; Haynes et al., 1989; Zulkifli Mahmoodin et al., 2019; Ortiz et al., 1992; Rippon & Brunswick, 2000; Spironelli et al., 2008; Taskov & Dushanova, 2021). Furthermore, alpha appears globally reduced in at rest conditions, whereas beta offers more contradictory results. If the theory of the compensatory beta effect is correct, it is possible that our younger samples do not exhibit compensatory effects yet.

The relationship between the deficits in different band frequencies and the stages of learning to read could also explain the scarcity of results in the gamma band of our review: It may be that the 12-year filter has determined a specific trend in the type of deficit. Interestingly, in our review, the alterations in the frequency bands appear to be associated with learning performance, supporting the neurobiological meaning of these components. It is noteworthy that when considering EEG frequency bands, it is important to consider that the bands may not be a perfect match with those of the adult or older child. Particularly, a shift in frequency peaks with age has been shown (Campus et al., 2021; Clarke et al., 2001; Orekhova et al., 2006).

The most heterogeneity in our review is in studies using stimulation. Most compared the EEG and the performance of DD and control children’s performance in linguistic, reading, or cognitive tasks. A smaller number of other studies evaluate several other tasks: writing, speech, spelling, music, listening to an audio story or someone reading, and tapping. The majority of tasks explore functions directly involved in dyslexia or strictly connected. However, there are also studies exploring different cognitive functions in DD children. These are interesting because they explore new hypotheses on dyslexia and its association with not obvious cognitive functions, like vigilance and visuospatial abilities. Unfortunately, the results are not comparable, given the different tasks and EEG analysis methodologies, but the differences between DD and control children appear mainly localized in the left temporoparietal sites. Still, caution should thus be taken in interpreting power differences between groups in the context of neural tracking differences, as they rely not only on distinct analytical approaches but also on different experimental paradigms. Probably for that reason, the data coming from our review does not capture the complex picture that emerges from the most recent research. For example, intriguing insights came from studies on the hemispheric specialization of specific frequencies in DD.

In summary, the left hemisphere appears to specialize in local high-frequency verbal computations, while the right hemisphere codes low frequencies of the speech envelope and interhemispheric cognitive control (Giraud & Poeppel, 2012). Impairment of the right hemisphere circuitry of frontoparietal attention networks has been hypothesized to be the primary cause of dyslexia (Goswami, 2011; Lehongre et al., 2011; Lizarazu et al., 2015; Molinaro et al., 2016; Power et al., 2016). Such a dysfunction would have a cascading negative effect on phonemic processing in the dorsal reading network (Kershner, 2019, 2020).

Our evaluation of the quality of the studies highlights an overall weakness of the reported studies. Many studies are old, and the methodological sections do not follow current guidelines of transparency and reliability of methods. The greatest weakness appears to be the small sample sizes of most studies; furthermore, almost none reported the method for selecting the sample size. Methodological concerns, the intrinsic high interindividual variability of electrophysiological techniques and the developmental phase, the tendency to publish only positive findings, and the use of different methodologies render the possibility of synthesizing and drawing conclusions very challenging. We could have included the grey literature to overcome these limitations, but we initially decided to limit the search to peer-reviewed published works. All these concerns hinder the possibility of establishing clear markers in EEG correlates of DD. However, a trend emerges despite differences in experimental conditions and analysis methodology (at rest, differences and involvement of the theta during reading-related tasks). The trend may reflect processing vulnerability in children with dyslexia or compensatory processing strategies that inappropriately activate areas of the reading network in this specific age range.

Finally, only a few studies evaluated the differences between dyslexic subtypes. A wide range of literature highlights the presence of different subtypes of DD. The few existing studies support differences at rest and during reading tasks. These conditions have to be better addressed because of the possible different cores (endophenotype) involved and the consequent additional variability in the results if not considered.

## Conclusion

This review seems to highlight some interesting insights: (a) there are abnormalities in spontaneous cerebral activity (“at rest”) of both temporal sites and more widerspread scalp placements in children with dyslexia, and (b) reading-related tasks elicited differences in frequencies considered crucial for speech processing, and the differences are localized in the temporoparietal sites. Although EEG localizations do not necessarily correspond to the underlying neuroanatomical regions, the finding of a left temporoparietal involvement is compatible with neuroimaging abnormalities, especially in the left and posterior regions. It should be noted that the current research on EEG correlates in DD is more advanced than is apparent from our review, which comprised a reduced number of works and some very old. This incongruency could denote a trend in research to select older participants, probably due to the greater simplicity of conducting studies with older and more collaborative children. Adolescents and adults are also suitable for more complex tasks. Furthermore, older age allows more certainty in diagnosis over time. However, we think that the range 6–12 is crucial because it represents the first appearance and diagnosis of the disorder and could offer important insights into the first phases of consolidation of both abilities and dysfunction. Therefore, we hope that future research addresses the functional role of atypical activation and involvement of specific frequencies in 6–12 years of DD to understand how fundamental brain processes are affected and how the brain compensates for those disruptions. Evaluation of the emergence and characterization of spectral EEG components and their deviation from the expected typical trajectory may be important to understanding early abnormalities of brain development, also in very early phases, as shown in the DD literature (Ozernov-Palchik & Gaab, 2016) and other research fields (Cainelli et al., 2021). This has the potential to lead to more effectiveness and could change the outcome trajectories for those with reading deficits.

## Supplementary Information

Below is the link to the electronic supplementary material.

Supplementary file1 (DOCX 20 KB)
