# Supplementary material for: EEG correlates of developmental dyslexia: a systematic review
Source: Ann Dyslexia. 2022 Nov 22;73(2):184–213. doi: 10.1007/s11881-022-00273-1 (PMC10247570; doi:10.1007/s11881-022-00273-1)
Supplement: Supplementary file 1 — Supplementary file1 (DOCX 20 KB) [file 11881_2022_273_MOESM1_ESM.docx]

S1. Quantitative EEG measures used in the reviewed articles.

| **Spectral measures** | |
| --- | --- |
| *Relative power and*  *absolute power* | Describes the distribution of power derived from the Fast Fourier Transform into the frequency components of which a signal is composed. Absolute power is the integral of the power within its frequency range in μV2, and relative band power can be calculated as an index over the total power. |
| *Asymmetry value* | Calculated as the difference between right and left power values in the brain regions of interest. |
| **Functional connectivity measures** | |
| *Coherence* | Coherence is the consistency between time series derived from two EEG channels normalized by their power spectra. |
| *Phase Lag Index* | The functional connectivity is calculated for all possible pairs of electrodes using the Phase Lag Index (PLI) separately for each frequency band between the two-time series. The PLI gives information about the phase synchronization of two signals by measuring the asymmetry of the distribution of their instantaneous phase differences. PLI does not depend on the signal's amplitude and is less sensitive to volume conduction in the brain and spurious correlations because of common sources. |
| *Complexity index* | The area under multiscale entropy curves; demonstrates structural  richness of information over multiple spatial and temporal scales.  Complexity gives insight into the adaptation of functioning and neuronal networks. |
| *Complexity* | The complexity of the EEG signal is measured by means of entropy:  the higher the complexity, the higher the entropy. |
| *Multiscale entropy* | The variability of EEG signals in long-range temporal dynamics. |
| *Minimum spanning tree* | The minimum spanning tree examines global brain connectivity and allows no recurrent connections, and networks are constructed with the weight adjacency matrices generated from the PLI analysis. Within the minimum spanning tree, all nodes are connected, but there is no recurrent connection. It focuses on connectivity strength; its topology network can be characterized by various metrics, e.g., degree, leaf fraction, diameter, eccentricity, betweenness centrality, Kappa, degree correlation, and tree hierarchy. |
| *Denoising autoencoder* | Autoencoders are a self-supervised neural network that is frequently used for feature extraction. It consists of a connection of an encoder and a decoder network, the former reducing the dimensionality of the input features x down to a bottleneck vector z of length len(z) len(x). Then, the output of the encoder is connected to a decoder network whose only purpose is to reconstruct the original signal ˆx using the information solely at z. The intermediate layer, or bottleneck, is commonly  known as z-layer. |
| *Directed transfer function* | Directed transfer function (DTF) provide directed connectivity between channels. To calculate the directed connectivity between a given channel A and a given channel B, this method estimates the ratio between the input from channel A to channel B and all the inputs to channel B. |
| **Localization** | |
| *Topographic maps* | In topographic mapping, EEG data are recorded from multiple scalp electrodes and graphically displayed on a computer-driven color video screen. Values between electrodes are obtained by interpolation. |
| *Loreta* | LORETA is a functional imaging technique belonging to a family of linear inverse solution procedures modeling 3D distributions of EEG sources. With respect to the dipole modeling of cortical sources, no a priori decision of the dipole position is required by LORETA, which belongs to the family of linear inverse algorithms like minimum norm solution, weighted minimum norm solution, or weighted resolution optimization. |
| *Vareta* | VARETA is a technique for estimating the primary current distribution in the source generators of EEG data. VARETA is a Discrete Spline Distributed Solution, like LORETA. The spline estimates are the spatially-smoothest solutions compatible with the observed data. VARETA, however, adapts to the actual degree of smoothness in each voxel being determined by the data itself by applying different amounts of spatial smoothing for different types of generators. |
